# Diagnostic performance of artificial intelligence approved for adults for the interpretation of pediatric chest radiographs

**DOI:** 10.1038/s41598-022-14519-w

**Published:** 2022-06-17

**Authors:** Hyun Joo Shin, Nak-Hoon Son, Min Jung Kim, Eun-Kyung Kim

**Affiliations:** 1 Department of Radiology, Research Institute of Radiological Science and Center for Clinical Imaging Data Science, Yongin Severance Hospital, Yonsei University College of Medicine, 363, Dongbaekjukjeon-daero, Giheung-gu, Yongin-si, Gyeonggi-do 16995 Republic of Korea; 2 Department of Statistics, Keimyung University, 1095, Dalgubeol-daero, Dalseo-gu, Daegu , 42601 Republic of Korea; 3 Department of Pediatrics, Institute of Allergy, Institute for Immunology and Immunological Diseases, Yongin Severance Hospital, Yonsei University College of Medicine, 363, Dongbaekjukjeon-daero, Giheung-gu, Yongin-si, Gyeonggi-do 16995 Republic of Korea

**Keywords:** Paediatric research, Respiratory tract diseases

## Abstract

Artificial intelligence (AI) applied to pediatric chest radiographs are yet scarce. This study evaluated whether AI-based software developed for adult chest radiographs can be used for pediatric chest radiographs. Pediatric patients (≤ 18 years old) who underwent chest radiographs from March to May 2021 were included retrospectively. An AI-based lesion detection software assessed the presence of nodules, consolidation, fibrosis, atelectasis, cardiomegaly, pleural effusion, pneumothorax, and pneumoperitoneum. Using the pediatric radiologist’s results as standard reference, we assessed the diagnostic performance of the software. For the total 2273 chest radiographs, the AI-based software showed a sensitivity, specificity, positive predictive value (PPV), negative predictive value (NPV), and accuracy of 67.2%, 91.1%, 57.7%, 93.9%, and 87.5%, respectively. Age was a significant factor for incorrect results (odds radio 0.821, 95% confidence interval 0.791–0.851). When we excluded cardiomegaly and children 2 years old or younger, sensitivity, specificity, PPV, NPV and accuracy significantly increased (86.4%, 97.9%, 79.7%, 98.7% and 96.9%, respectively, all p < 0.001). In conclusion, AI-based software developed with adult chest radiographs showed diagnostic accuracies up to 96.9% for pediatric chest radiographs when we excluded cardiomegaly and children 2 years old or younger. AI-based lesion detection software needs to be validated in younger children.

## Introduction

Recently, artificial intelligence (AI) has been widely adopted for medical imaging, for the segmentation, detection, diagnosis of lesions and even for prognosis prediction. Among various imaging tools, chest radiographs are one of the most commonly assessed modalities using AI^1^. This is because chest radiographs are frequently collected in daily practice to help guide the initial steps of patient management. In addition, chest radiographs can be used to detect critical and emergent diseases. However, obtaining a radiologist’s radiograph report is not easy in a time-pressed situation and clinicians encounter urgent situations requiring prompt decisions based on their own interpretations of chest radiographs. Therefore, there is a clinical need for AI-based lesion detection software for chest radiographs.

Various studies have demonstrated the potential and performance of AI-based lesion detection software on adult chest radiographs, with reports documenting its use for the detection of pneumonia, pneumothorax, lung nodule, tube positioning, and for COVID-19^2–7^. However, reports in which AI-based methods have been applied to pediatric chest radiographs are yet scarce, and most studies were performed in the earlier era of pediatric radiology for orthopedic and body X-ray images^8,9^. Chest radiographs still play a critical role in pediatric radiology with even more importance than in adults, because advanced imaging studies cannot be freely performed in children. In addition, frequently affected intrathoracic diseases of pediatric patients, such as infection, usually first appear on chest radiographs and most of the thoracic diseases seen in adults, such as pneumothorax and pleural effusion, can also affect children. This means the clinical necessity of the AI-based approach is no less for children than it is for adults.

Therefore, this study aimed to evaluate whether AI-based lesion detection software that was developed and approved for adult chest radiographs could be used for pediatric chest radiographs. We wanted to know the clinical potential of an AI-based solution for pediatric chest radiographs and to identify specific age groups for which the software needs further validation before clinical application.

## Results

During the study period, a total of 2273 chest radiographs (M:F = 1280:993, mean age 7.0 ± 5.8 years old, range 0–18 years old) were included for analysis. Among them, 347 radiographs (15.3%) had positive results when assessed by the radiologist.

### Diagnostic performance including all lesions

**Figure 1:**
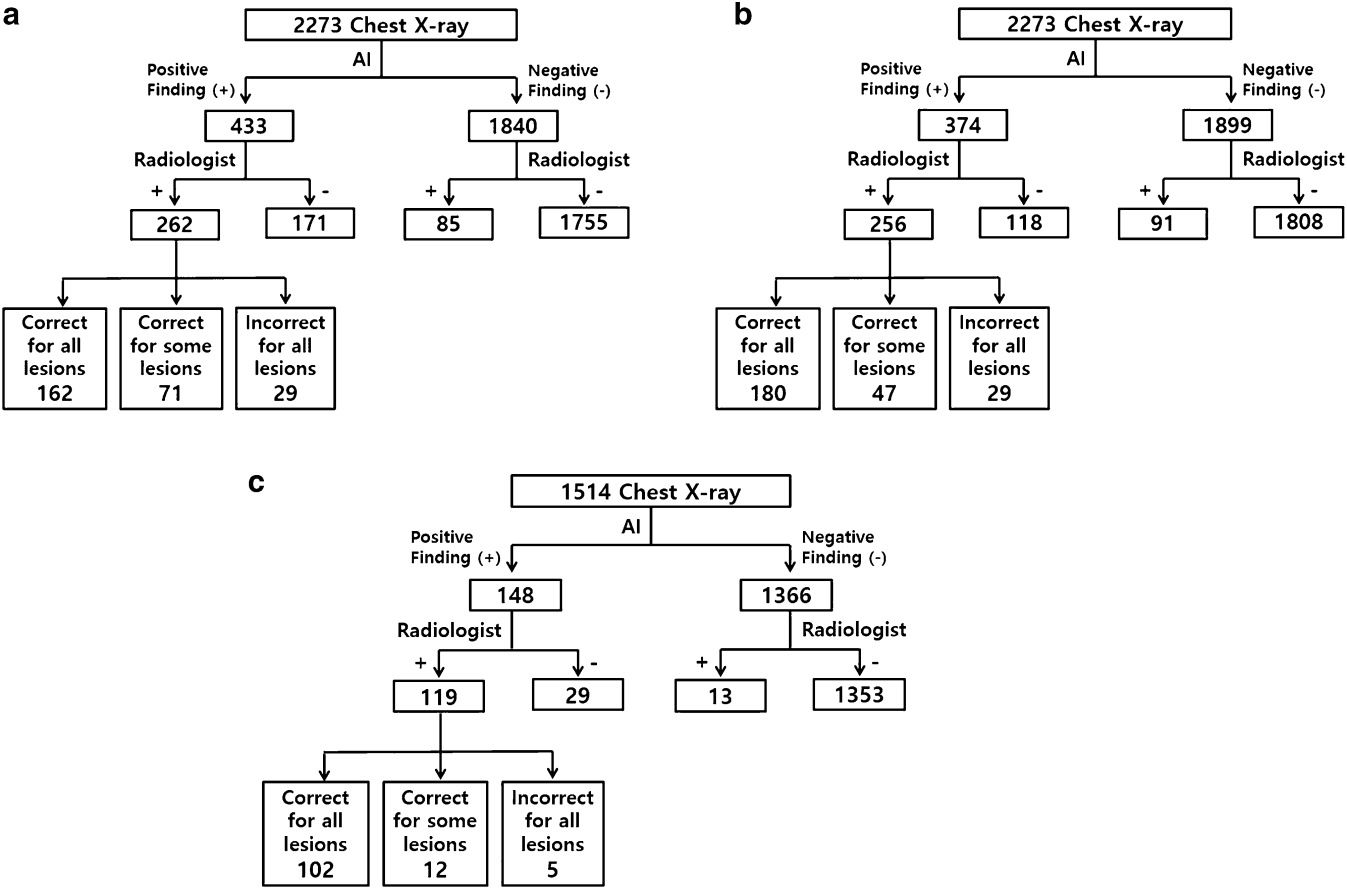
Flowcharts of diagnosis including (**a**) all lesions, (**b**) excluding cardiomegaly from all lesions, and (**c**) excluding cardiomegaly and patients ≤ 2 years old.

When we included all eight types of detectable lesions (nodules, consolidation, fibrosis, atelectasis, cardiomegaly, pleural effusion, pneumothorax, and pneumoperitoneum) in subset (A), the AI-based software assessed 433 radiographs (19.1%) as positive and 1840 radiographs as negative (Fig. 1A). Among the 433 positive radiographs, the radiologist assessed 171 radiographs as negative, implying these were false-positive results. Of the remaining 262 positive radiographs, 162 radiographs were correct for all lesions and 71 radiographs were correct for some lesions. When we considered the results also assessed as positive by the radiologist, 233 radiographs were true-positive images. The remaining 29 images were actually incorrect for all lesions and were false-negative results. Among the 1840 images assessed as negative by the software, 1755 radiographs had no lesions and this implied true-negative results. The other 85 negative images were assessed as positive by the radiologist and were considered as false-negative images. Therefore, the sensitivity, specificity, PPV, NPV and accuracy of the software was 67.2%, 91.1%, 57.7%, 93.9%, and 87.5%, respectively (Table 1).

**Table 1:** Comparison of diagnostic performances of the AI-based software for subsets (A)–(C).

	Sensitivity	p-value	Specificity	p-value	PPV	p-value	NPV	p-value	Accuracy	p-value
(A) Including all lesions	67.2 (62.2–72.1)	–	91.1 (89.9–92.4)	–	57.7 (52.9–62.5)	–	93.9 (92.8–95)	–	87.5 (86.1–88.8)	–
(B) Excluding cardiomegaly	65.4 (60.4–70.4)	0.014^¶^	93.9 (92.8–94.9)	< 0.001^¶^	65.8 (60.8–70.8)	< 0.001^¶^	93.8 (92.7–94.9)	0.310^¶^	89.5 (88.3–90.8)	< 0.001^¶^
(C) Excluding cardiomegaly and children ≤ 2 years old	86.4 (80.5–92.2)	< 0.001^¥^	97.9 (97.1–98.7)	< 0.001^¥^	79.7 (73.1–86.3)	< 0.001^¥^	98.7 (98.1–99.3)	< 0.001^¥^	96.9 (96.0–97.8)	< 0.001^¥^

Values are presented in percentages (%) with 95% confidence intervals.

^¶^Comparison between subset (A) and (B). ^¥^Comparison between subset (B) and (C).

*CI* confidence interval, *PPV* positive predictive value, *NPV* negative predictive value.

The diagnostic performance of the AI-based software for each type of lesion is presented in Table 2 to show how effective the software was for critical cases. Fibrosis and pneumoperitoneum were excluded because of their small incidence (three for fibrosis, none for pneumoperitoneum). Among the other lesions, the software showed a sensitivity of 98.5%, specificity of 99.6%, and accuracy of 99.6% for pneumothorax.

**Table 2:** Diagnostic performance according to each lesion type.

Lesion (number of cases)	Sensitivity (%)	Specificity (%)	PPV (%)	NPV (%)	Accuracy (%)
Nodule (n = 16, 0.7%)	93.8 (69.8–99.8)	96.6 (95.8–97.3)	16.5 (13.3–20.3)	99.9 (99.7–99.9)	96.6 (95.8–97.3)
Pneumothorax (n = 67, 3%)	98.5 (92.0–99.9)	99.6 (99.3–99.8)	89.2 (80.5–94.3)	99.9 (99.7–99.9)	99.6 (99.3–99.8)
Consolidation (n = 238, 10.5%)	63.9 (57.5–70.0)	96.4 (95.5–97.1)	67.3 (61.7–72.4)	95.8 (95.1–96.4)	93.0 (91.8–94.0)
Atelectasis (n = 18, 0.8%)	55.6 (30.8–78.5)	99.8 (99.5–99.9)	66.7 (43.2–84.0)	99.7 (99.4–99.8)	99.4 (99.0–99.7)
Cardiomegaly (n = 20, 0.9%)	90 (68.3–98.8)	94.0 (92.9–94.9)	11.7 (9.6–14.1)	99.9 (99.5–99.9)	93.9 (92.9–94.9)
Pleural effusion (n = 23, 1%)	69.6 (47.1–86.8)	99.5 (99.1–99.8)	59.3 (43.2–73.6)	99.7 (99.4–99.8)	99.2 (98.8–99.5)

Values are presented with 95% confidence intervals.

*PPV* positive predictive value, *NPV* negative predictive value.

### Diagnostic performance excluding cardiomegaly from all lesions

When we excluded cardiomegaly from all lesions for subset (B), the AI-based software assessed 374 radiographs (16.5%) as positive and 1899 radiographs as negative (Fig. 1B). Among the 374 positive radiographs, the radiologist assessed 118 radiographs as negative, implying false-positive results. Among the other 256 positive radiographs, 180 were correct for all lesions and 47 were correct for some lesions. When we consider images also assessed as positive by the radiologist, 227 radiographs were true-positive images. The remaining 29 images were actually incorrect for all lesions when the radiologist’s result was considered the ground truth and were thus false-negative images. Among the 1899 radiographs assessed as negative by the software, 1808 had no lesions and this implied true-negative results. The remaining 91 images were positive for lesions when assessed by the radiologist and were considered false-negative images.

Therefore, the sensitivity, specificity, PPV, NPV and accuracy of the software was 65.4%, 93.9%, 65.8%, 93.8%, and 89.5%, respectively. When we excluded the cardiomegaly results, specificity, PPV and accuracy significantly increased (all, p < 0.001). Sensitivity significantly decreased (p = 0.014), while NPV showed no significant difference (p = 0.31) (Table 1).

**Figure 2:**
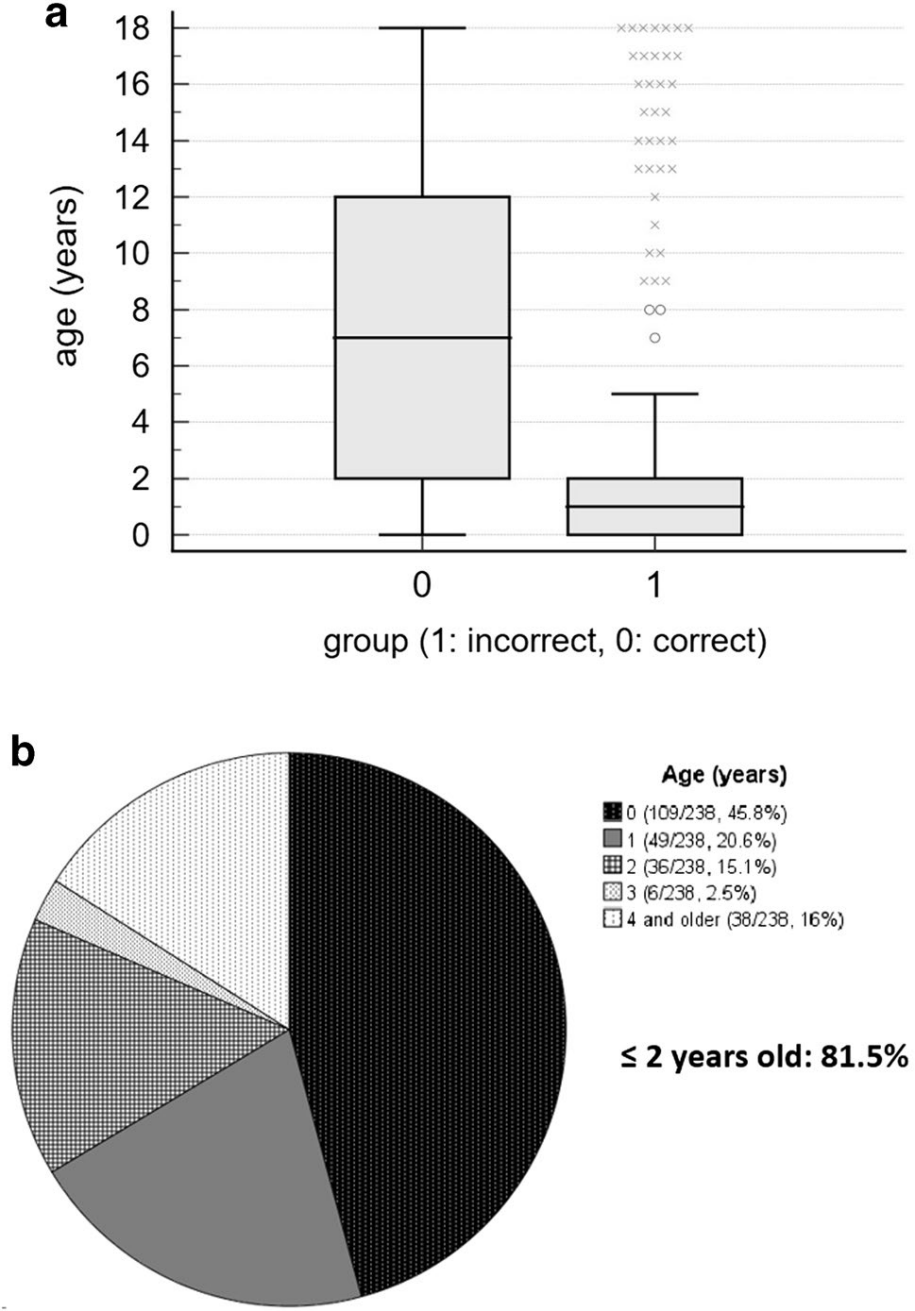
Age distribution for correct and incorrect diagnoses after excluding cardiomegaly. (**a**) Box-whisker plot comparing the median, interquartile ranges and entire range of age according to diagnosis. (**b**) Pie chart depicting the age distribution of the incorrect diagnosis group.

When we compared age between patients with correct and incorrect diagnoses in subset (B), the age of patients with incorrect diagnosis was significantly younger than those with correct diagnosis (median 1 year vs. 7 years, p < 0.001) (Fig. 2A). In the logistic regression test, age was a significant factor for incorrect diagnosis (odds ratio 0.821, 95% confidence interval 0.791–0.851, p < 0.001). In the 238 patients with incorrect diagnosis, age distribution was as follows; 45.8% in patients less than 1 year of age, 20.6% 1 year, 15.1% 2 years, 2.5% 3 years, and 16% 4 years or more (Fig. 2B). Therefore, patients 2 years old or younger occupied 81.5% of patients with incorrect diagnosis.

### Diagnostic performance excluding cardiomegaly and patients ≤ 2 years old

Subset (C) was defined with results obtained when children 2 years old or younger were excluded after excluding the cardiomegaly results. In this subset, the AI-based software assessed 148 among 1514 radiographs (9.8%) as positive and the remaining 1366 as negative (Fig. 1C). The sensitivity, specificity, PPV, NPV and accuracy was 86.4%, 97.9%, 79.7%, 98.7% and 96.9%, respectively. The diagnostic performances for subset (C) were all significantly increased compared with those for subset (B) (all, p < 0.001, Table 1).

## Discussion

An AI-based approach has been widely developed and studied for medical imaging, especially for chest radiology^10^. Many studies have validated the diagnostic performances of AI-based software and its potential effects on the clinical process using commercially available AI-based solutions for the chest radiographs of adults^7,11–13^. A few recent studies have tried to use AI-based algorithms for pediatric chest radiographs. Salehi et al. demonstrated diagnostic accuracies up to 86% using chest radiographs for the binary classification of pneumonia in children younger than 5 years old with transfer learning^14^. In another study on pneumonia and pleural effusion, both AI and radiologists showed similar diagnostic performances; about 74–78% for pneumonia and 70–74% for pleural effusion^15^. Another pneumonia study subdivided radiologic patterns on pediatric chest radiographs and AI showed an accuracy of 81% for diagnosing definite pneumonia^16^. Apart from disease detection, Moore et al. tried to predict disease severity using AI on pediatric chest radiographs^17^. Their AI-based model predicted the severity score of cystic fibrosis using chest radiographs and found a maximum correlation coefficient of 0.83 compared to the Brasfield scores of radiologists^17^. However, very few studies have utilized commercially available software with sufficient diagnostic performance for variable lesions on pediatric radiographs. Several researchers have tried to adopt adult-oriented AI concepts for pediatric imaging, but this research is still in early stages^8,18^. Others have also predicted the potential utilization of adult-based AI methods in pediatric imaging, but contrary to expectations, one report showed inadequate results for diagnosing vertebral fracture using such software^8,19^. Our study is the first attempt to validate adult-oriented software for pediatric chest radiographs and this is considered to have clinical value because chest radiographs are widely utilized in children.

Our study showed that commercially available AI-based lesion detection software had an accuracy of 87.5% when eight types of lesions were analyzed, and 89.5% when only cardiomegaly was excluded. Patients with incorrect diagnosis were significantly younger than patients with correct diagnosis, and patients 2 years old and younger occupied 81.5% of the incorrect results. When we excluded children ≤ 2 years old, accuracy increased up to 96.9% and this result was comparable to the diagnostic accuracy provided by the vendor for adults (97–99%)^20^. Even though the diagnostic performance of this software was not fully analyzed for all eight lesions through external validation even in adults, its performance was widely discussed^21,22^. Therefore, our results indicate that comparable performance can be achieved in children. This suggests that more qualified data of younger children are needed before adult-based AI software can be applied to pediatric chest radiographs and that adult-based AI software might show excellent diagnostic performance for detecting specific lesions in older children. In addition, among the detectable lesions, AI software showed the highest accuracy for pneumothorax. Chest disease can differ between children and adults and the clinical impact and incidence of each type of lesion, such as fibrosis, can be different. The detection of critical cases with high diagnostic performance is another issue of importance for children. This result showed that AI-based solutions can be used to screen critical cases and this could raise its clinical significance. Our results demonstrated the potential utilization of an adult-oriented AI algorithm for lesion detection with specific ages and disease entities taken into consideration. Our results show that efforts have to be made to develop pediatric-specified AI-based software with qualified data. Adult-oriented AI-based software can be a starting point for this as images of a specific age range can be added to previously existing software or by indicating the type of lesion for which the developed software requires more validation.

This study had several limitations. First, a spectrum bias could exist due to disease severity and prevalence because data were collected from a single hospital. However, we tried to include all consecutive data during the study period to avoid selection bias. In addition, because hospitals of various sizes could have patients with different disease characteristics, it is meaningful to assess and validate our findings in diverse clinical settings. Second, using a single commercially available software could be a limitation of our study, despites its performance being approved through several certified peer-reviewed journals^1,21,22^. Using different software programs could result in different degrees of robustness when each software is applied to pediatric chest radiographs and this variation needs to be addressed in a future study. Third, we used a preset operating point of 15% to determine the presence of lesions. This operating point of 15% may not be appropriate for other lesion types and pediatric conditions, suggesting that further research will need to focus on this subject to set effective cutoff values. Finally, the radiograph interpretation of one pediatric radiologist was used as the gold standard. The experienced radiologist diagnosed the radiographs with all available software results, clinical information and comparable images to avoid misinterpretations. However, our study is a first validation study and future studies should focus on whether the AI-based solution can change the decision-making process for pediatric radiographs and whether it can find lesions that radiologists cannot detect. In addition, further multi-center studies with diverse patient populations are needed for accurate validation.

In conclusion, when AI-based lesion detection software developed with adult chest radiographs was applied to children, it showed diagnostic accuracies up to 96.9% when cardiomegaly and children 2 years old and younger were excluded, suggesting that AI-based lesion detection software can be developed and utilized for pediatric chest radiographs after further validation. AI-based lesion detection software needs to be validated in younger children with larger data to assure safe usage, and adult-oriented software can be a starting point for this.

## Methods

### Subjects

The Institutional Review Board (IRB) of Yongin Severance Hospital approved this retrospective study (IRB number 9-2021-0089). The need for informed consent was waived by the IRB of Yongin Severance Hospital due to retrospective nature of the study. All methods were carried out in accordance with Strengthening the Reporting of Observational Studies in Epidemiology (STROBE) guideline and regulations. All methods were performed in accordance with relevant guidelines and regulations. This study was performed in accordance with the Declaration of Helsinki. Pediatric patients (≤ 18 years old) who underwent chest radiographs from March to May 2021 were included in this study. Posteroanterior (PA) and anteroposterior (AP) views of the chest radiographs were included for analysis, while lateral and decubitus chest radiographs were excluded.

### AI-based lesion detection software

A commercially available, Conformité Européenne (CE)-certified AI-based lesion detection software (Lunit INSIGHT for Chest Radiography, version 3, Lunit Inc, Korea) that was solely developed and approved for adult chest radiographs was used in this study^22,23^. The used algorithm was a ResNet34-based deep convolutional neural network that was recently approved for lesion detection including the detection of nodules, consolidation, fibrosis, atelectasis, cardiomegaly, pleural effusion, pneumothorax, and pneumoperitoneum^1,6,21,22^. The AI algorithm included both PA and AP views of chest radiographs during training of the model. The labels such as AP, PA annotations were included without deletion during preprocessing of algorithm. The intended usage was for patients 19 years old or older.

**Figure 3:**
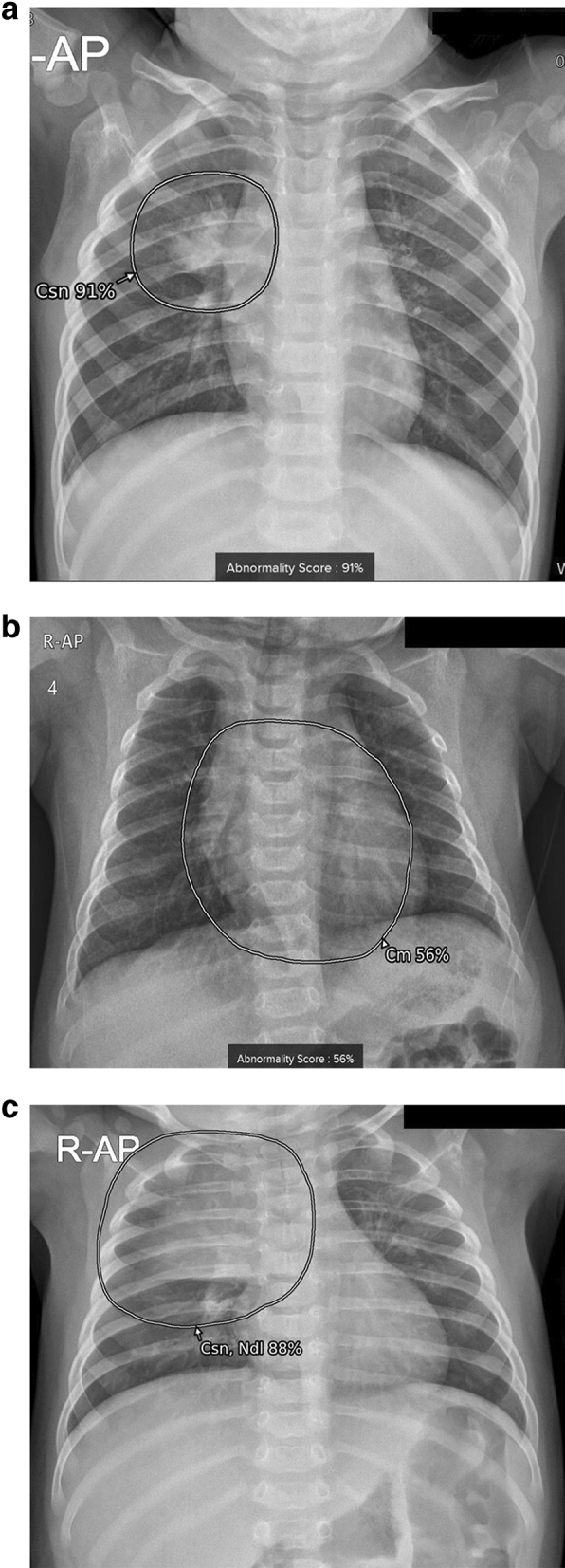
Examples of results analyzed by the AI-based lesion detection software. (**a**) A 17-month-old boy with pneumonia in the right upper lobe. The software detected consolidation (Csn) with an abnormality score of 91% in the right upper lobe, as marked in the grayscale map. (**b**) A 3-month-old boy with a cardiothoracic ratio of 50%, within normal range. The software detected cardiomegaly (Cm) with an abnormality score of 56% on the anteroposterior chest radiograph. (**c**) A 4-month-old girl without remarkable findings on the chest radiograph. The software detected normal thymus as consolidation (Csn) and nodule (Ndl) with an abnormality score of 88%.

The standard Digital Imaging and Communications in Medicine (DICOM) file formats were used for analysis without restrictions from the device manufacturer. After the analysis, suspicious areas of each lesion were presented with a grayscale map and abnormality scores were calculated and presented automatically in the secondary capture image (Fig. 3). The probability for the presence of suspicious areas was provided in percentages. When the calculated score was above the preset operating point of 15%, the region and abnormality score of the area in question appeared on images. This operating point was provided by vendor for detecting abnormalities on adult chest radiographs. The total abnormality score per image was presented by depicting the highest score of each lesion in the image.

### Image analysis by a radiologist

A board-certified pediatric radiologist with 11 years of experience evaluated each chest radiograph for lesions. Among the commercially approved lesion types, a total of eight which were nodules, consolidation, fibrosis, atelectasis, cardiomegaly, pleural effusion, pneumothorax, and pneumoperitoneum were analyzed on the pediatric radiographs. The radiologist confirmed each abnormality on images and then each lesion in reference to the software results. The radiologist decided to accept or reject the software results after this review. During this process to confirm lesions, patient information such as age, clinical data and results from other available imaging studies were provided to the radiologist as much as possible to minimize false decisions.

### Statistical analysis

Statistical analyses were performed using SAS software version 9.4 (SAS Institute Inc., Cary, NC, USA) and SPSS version 25 (IBM Corp., Armonk, NY, USA). With the radiologist results considered the gold standard, we assessed the diagnostic performance of the AI-based software. Sensitivity, specificity, positive predictive value (PPV), negative predictive value (NPV) and accuracy were evaluated. The diagnostic performance of the software was evaluated in three subsets; (A) for all lesions, (B) all lesions but excluding cardiomegaly, and (C) all lesions but excluding cardiomegaly and those from specific age groups with incorrect results. When we analyzed all lesions, diagnostic performances were assessed according to each type of lesion to determine the clinical implication of the AI-based software for critical cases, such as pneumothorax, among the detectable lesions. After that, for subset (B), we assessed secondary diagnostic performances after excluding cardiomegaly, because different criteria are used to diagnose cardiomegaly in adults and young children^24^. For example, cardiomegaly can be diagnosed by radiologists using the cardiothoracic (CT) ratio on chest radiographs, with the normal CT ratio range being below 60% for neonates and below 50% for adults. For subset (C), we assessed which age was mostly affected by incorrect diagnoses of false-positive and false-negative results in lesions from subset (B). To compare age between patients with correct and incorrect diagnoses, the Mann–Whitney *U* test was used after the Kolmogorov–Smirnov test. The logistic regression test was used to assess the effect of age on incorrect diagnoses. A generalized estimating equation was used to compare the diagnostic performances of the software for the subsets. P-values less than 0.05 were considered statistically significant.

## Data Availability

The datasets generated or analyzed during the study are available from the corresponding author on reasonable request.

## Abbreviations

AI: Artificial intelligence
PPV: Positive predictive value
NPV: Negative predictive value
CI: Confidence interval
